# Physical activity patterns among obese adults attending rural primary health care units, Ismailia Governorate, Egypt: A case-control study

**DOI:** 10.1097/MD.0000000000037328

**Published:** 2024-03-01

**Authors:** Almaza A. Salim, Eman Fahmey Nasr, Yomna E. Dean, Jose J. Loayza Pintado, Noha M. Abu Bakr Elsaid, Yusef Hazimeh, Mostafa M. Ragheb, Hani Aiash

**Affiliations:** a Family Medicine Department, Faculty of Medicine, Port Said University, Egypt; b Internal Medicine Department, Faculty of Medicine, Suez Canal University, Egypt; c Faculty of Medicine, Alexandria University, Egypt; d Alexandria Medical Center (AMC), Alexandria, Egypt; e Universidad de San Martín de Porres Facultad de Medicina Humana, Peru; f Department of Public Health, Community, Environmental and Occupational, Faculty of Medicine, Suez Canal University, Egypt; g Department of Basic Medical Sciences, Faculty of Medicine, King Salman International University, South Sinai, Egypt; h Lebanese University, Beirut, Lebanon; i SUNY Upstate Medical University, Syracuse, NY.

**Keywords:** obesity, physical activity, recreational activities

## Abstract

Obesity is an ignored health problem in all countries; there are a lot of health problems related directly or indirectly to overweight and obesity. The incidence of COVID-19 with social isolation and technological development in recent years strongly contributed to a progressive increase in obesity. . Assess the pattern of the 3 divisions of physical activity and sedentary behaviors in obese patients. Physical inactivity is a significant concern, especially among individuals with obesity and certain demographic characteristics. Addressing these factors and promoting physical activity interventions tailored to specific populations is essential in combating sedentary behavior and its associated health implications.This case-control study included 350 adult obese patients (BMI ≥ 30) and 75 people with normal BMI (18.5–24.9). Their sociodemographic data were analyzed and their pattern of physical activity related to work, movement to and from places for 10 minutes, and pattern of recreational activity were assessed, in addition to the assessment of the sedentary behaviors. The mean age of the study group was 34 years, the majority were females, educated, and working. Forty five percent of the total sample were physically inactive; the pattern of activity during travel to and from places (10 min) was lower in obese patients. Recreational activities were low in the studied population, in the present study the time spent sitting or reclining (except sleeping) was significantly higher among obese participants than controls (*P* ≤ .001). Obesity, urban residence, unemployment and illiteracy were independent risk factors for physical inactivity.

## 1. Introduction

Obesity is characterized by an abnormal accumulation of fat, posing significant health risks, particularly concerning cardiovascular diseases and type 2 diabetes, which are major contributors to premature mortality. The escalating prevalence of obesity worldwide has become a pressing issue.^[1]^ In fact, obesity is responsible for the deaths of over 3 million people, making up approximately 4% of the leading global risks for disease burden, as measured by years of life lost and disability-adjusted life years.

There are a lot of health problems caused by obesity such as hypertension and hypertensive-related heart diseases, pulmonary diseases such as asthma and obstructive sleep apnea, diabetes mellitus (DM), orthopedic problems such as back ache and osteoarthritis, and multiple different types of cancers like colorectal, pancreatic, prostate and some cancers related to females such as breast, ovarian, and endometrial carcinomas which consequently increase the prevalence of morbidity and mortality.^[2]^

Multiple studies showed that obesity is a reason for a lot of public health diseases that consequently results in decreased life expectancy. The body mass index (BMI) by itself without other anthropometric measures is a strong predictor of morbidity and mortality.^[3]^ Chronic health problems related to obesity cause a significant impact on expenditure of health and the countries at all.^[2]^

Obesity is associated with a number of diseases that can weaken the immune system, making obese individuals more vulnerable to infectious diseases such as COVID-19.^[4]^ Additionally, the effectiveness of vaccines tends to be lower in overweight and obese patients.^[5]^ The global prevalence of obesity is on the rise, partially influenced by the COVID-19 pandemic, with Middle Eastern countries showing the highest rates.^[6]^ Egypt, for instance, has an obesity prevalence of approximately 36%, with higher occurrences among individuals in low socioeconomic conditions compared to those in high socioeconomic statuses.^[7]^

The emergence of COVID-19 led to various consequences, including movement restrictions, limited social interactions, and economic downturns. As a result, there have been notable impacts on food consumption and lifestyle changes, particularly concerning physical activity, diet, and increased engagement in online activities. These factors, in turn, have contributed to a rise in the obesity rate.^[8]^

Engaging in physical activity yields beneficial health effects on various body organs, including the cardiovascular and cerebrovascular systems. It acts as a preventive measure against numerous diseases, such as various cancers, DM, and anxiety. Moreover, physical activity enhances cognitive functions, boosts the capacity to acquire new skills, and contributes to overall well-being and improved health.^[9]^

Physical activity encompasses all body movements during the day, whether they occur while working, commuting (e.g., walking, cycling, or using a wheelchair), or during leisure time (playing, engaging in sports, games, or planned exercises). According to the World Health Organization (WHO), adults aged 18 to <65 years should aim for 150 to 300 minutes of moderate-intensity aerobic physical activity or 75 to 150 minutes of vigorous-intensity activity each week. Alternatively, they can combine both types of activities to meet the criteria for being considered physically active. Conversely, individuals who do not meet these criteria are classified as physically inactive.^[10]^

Physical inactivity significantly raises the risk of developing multiple diseases and mortality. Inactive individuals face a 20% to 30% higher risk of death compared to their physically active counterparts.^[11]^ Unfortunately, there is limited understanding of physical activity patterns, particularly in rural and low socioeconomic populations, where obese patients are particularly susceptible to various preventable conditions.

For this reason, our study aimed to examine the physical activity patterns and sedentary behavior of obese patients in comparison to individuals with a normal BMI attending a rural primary health care unit.

## 2. Methodology

This is a case-control study for the assessment of the physical activity pattern in obese patients attending El-Mahasma rural primary health care in Ismailia governorate, Egypt. The study population included 350 obese (BMI ≥ 30 kg/m^2^) and 75 healthy individuals (BMI 18.5–24.9 kg/m^2^); with a total sample size of 425. The case group was composed of individuals who met the following criteria: BMI > 30 kg/m^2^, age over 18 years, and both genders. We did not exclude participants with comorbidities related to obesity (such as diabetes, hypertension, ischemic heart disease, hypothyroidism, or chronic obstructive pulmonary disease). However, we excluded patients who suffered from psychiatric illnesses and/or physical disabilities.

## 3. Sample calculation

The sample had been determined by the following equation:

n = (*Z*/SE)² × p × (1 − p).^[12]^

n = sample size

p = prevalence of physical inactivity among Egyptian adults in 2016 was 31%^[13]^

*Z* = percentile of standard normal distribution determined by 95% confidence level = 1.96%, SE = 0.05.

Sample size (n) = (1.96/0.05)² × 0.31 (1 − 0.31) = 329 patients. After adding 5–10% nonresponse rates, the total number was 350 obese patients, besides 75 individuals with normal BMI which had been taken as the control group, having 425 participants as the total sample.

Until the necessary data collection was finalized, none of the obese patients visiting El-Mahsma primary health care unit were included in the research. The data was gathered through interviews using a validated and structured questionnaire. This questionnaire was the Arabic version of the WHO Global Physical Activity Surveillance, comprising 2 sections.

The first section focused on patients’ demographics, BMI measurements, and clinical examinations to determine the severity of obesity. The second section delved into assessing their physical activity levels concerning workplace activities, transportation to and from places, leisure activities, and sedentary behavior.^[14]^

The World Health Organization (WHO) defines being physically active as engaging in at least 150 minutes of moderate physical activity or 75 minutes of vigorous physical activity per week, or a combination of both 10. On the other hand, the Sedentary Behavior Research Network classifies a sedentary lifestyle as spending more than 6 h/d sitting without engaging in any physical activity.^[15]^

## 4. Management of data

The data were analyzed using IBM SPSS software package version 20 (Armonk, NY: IBM Corp). The Kolmogorov–Smirnov test was used to verify variable distribution. Descriptive results examine the percentage of time spent in each domain relative to the overall level of physical activity. Comparisons between groups for categorical variables were assessed using the Chi-square test (Monte Carlo). The Kruskal–Wallis test was used to compare more than 2 categorical variables. *P* value was analyzed to determine the significance of the results. Logistic regression was conducted to determine the risk factors of physical inactivity.

## 5. Results

The study population included 425 individuals, 350 patients diagnosed with obesity, and 75 individuals in the control group; the mean age was 34 years in both groups. Two-thirds of the study group and more than half of the control were females, the majority was married, about three-quarters were educated and more than half of them were working. About 13% of the studied group has a past medical history of COVID-19 while 36% of the control had COVID-19 (Table 1).

**Table 1:** Demography of the studied population (N = 425).

Characteristics	Cases (n = 350)	Control (n = 75)	*P* value
Gender			
Male	76 (21.7%)	33 (44.0%)	<.001
Female	274 (78.3%)	42 (56.0%)	
Age			
<40	269 (76.9%)	51 (68.0%)	.107
>40	81 (23.1%)	24 (32.0%)	
Mean ± SD	34.22 ± 9.30	34.91 ± 7.30	.191
Median (Max–Min)	34 (18.0–67.0)	35 (19.0–53.0)	
Residence			
Rural	302 (86.3%)	64 (85.3%)	.829
Urban	48 (13.7%)	11 (14.7%)	
Marital status			
Married	276 (78.9%)	63 (84.0%)	p = .712
Unmarried	74 (22.1%)	12 (16%)	
Special habits			
Smoker	51 (14.6%)	13 (17.3%)	.544
Nonsmoker	299 (85.4%)	62 (82.7%)	
Occupational history			
Working	203 (58%)	52 (69%)	p = .318
Not working	147 (42%)	23 (31%)	
Educational level			
Educated	251 (72%)	56 (75%)	.002
Uneducated	99 (18%)	19 (25%)	
History of COVID-19			
Yes	48 (13.7%)	27 (36.0%)	<.001
No	302 (86.3%)	48 (64.0%)	
Total	350 (82.3%)	75 (17.6%)	

MC = Monte Carlo, p = *P* value for comparing between the studied groups.

**Figure 1.**
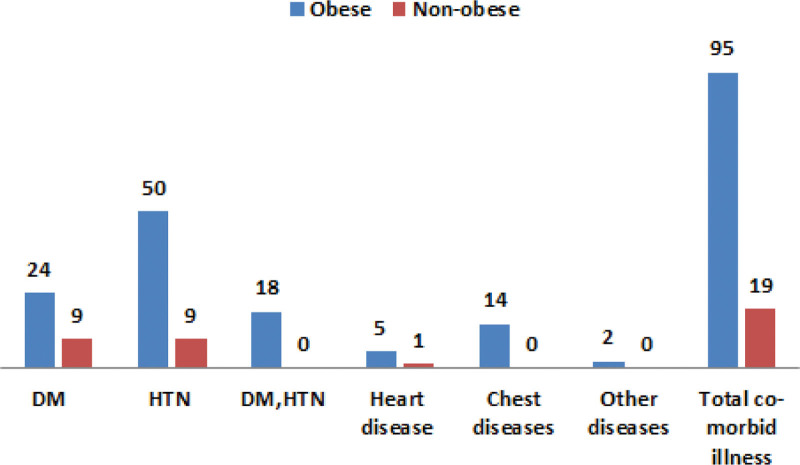
The distribution of comorbid diseases in the studied population.

**Figure 2.**
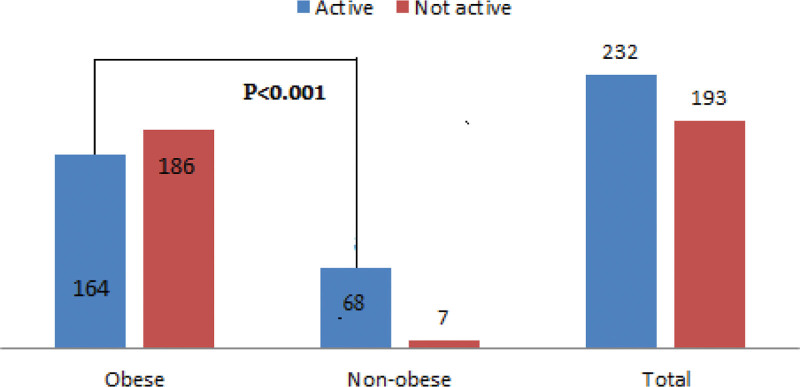
The distribution of obese versus nonobese participants according to the physical activity pattern.

Regarding presence of comorbid diseases, about 27% of study group individuals compared to 21% in the control group had comorbid disease, the most common disease was hypertension (14%, 12%), chest disease and DM. However, the difference in both groups was not statistically significant (Fig. 1 and Table 2). Regarding diseases, in the studied population, 95 out of 350 obese patients had comorbid disease, compared to 19 out of 75 in nonobese participants in whom hypertensive diseases were the most common diseases followed by DM and chest disease. Regarding physical activity, about 45% of the total sample was physically inactive. In the case group, above half (53.5%) were physically inactive compared to a 9% in the control group. The difference between the 2 groups was statistically significant (*P* < .001) as shown in Table 3. Figure 2 shows that physical activity was significantly more frequent among attendants with average body build compared to obese ones (*P* < .001). Although the pattern of vigorous physical activity at work was more in the studied group compared to control one (26% vs 20%), it was statistically insignificant; the mean number of days per week were less in cases compared to controls and being statistically significant (3.32 ± 1.77 in the cases group, 5.13 ± 1.19 in control group, *P* < .001). Also, the mean number of minutes of vigorous activity per day in the study group were less than the control 1 with a statistically significant result (296.7 ± 120.5, 380.0 ± 95.3, and *P* = .005). Regarding the pattern of moderate physical activity related to work, about two-third of cases compared to one-quarter of the control group were not active, the mean number of days per week were less in the case group than in the control group with statistically significant value (3.38 ± 1.74, 4.87 ± 1.33, *P* < .001). In addition, the mean number of minutes of moderate activity per day in the study group was less than the control 1 with a statistically significant value (261.8 ± 120.4, 355.6 ± 112.5, *P* < .001) as in Table 4. Regarding pattern of activity during travel from and to places, 50% of obese patients practice walking from and to places more than 10 minutes during the day compared to 84% in the control group with a significant *P*-value (*P* = <.001); the mean number of days per week 1.65 ± 1.28 in the case group compared to 2.56 ± 1.47 in the control 1, and the mean number of minutes per day were 86.40 ± 50.88 in the case group and 129.5 ± 85.67 in the control group which was statistically significant (*P* < .001; Table 5). The pattern of leisure or fun activities was less frequent in cases compared to controls. The frequency of vigorous activity among cases was 53 (15%) compared to 7 (9%) in the control group, however, it was statistically insignificant except in the mean number of minutes per day where it was 100.8 ± 34.9 in cases and 171.4 ± 80.71 in controls with *P*-value = .015. The frequency of moderate-intensity activity among cases was 3% compared to 10% in the control group with an statistically significant *P*-value (*P* = .014), the mean number of days per week (3.25 ± 1.29 and 2.63 ± 1.51) and the mean number of minutes per day (215.0 ± 90.3 and 135.0 ± 62.1) was statistically insignificant as in Table 6. Regarding the pattern of sedentary behavior, in our study more than 50% of patients (202 out of 350) spent more than 6 h/d in sitting or reclining position compared to a 30% in the control group (24 out of 75) with a significant *P*-value (*P* < .001) as in Table 7. By doing logistic regression analysis for detection of factors affecting physical activity in the total sample, it was found that obese patients, females, persons who were resident in urban areas and persons who were unemployment were more physically inactive compared to male genders, with normal range BMI, live in rural areas and employed (odds ratio [OR] = 11 [4.9–24.6], OR = 1.6 [1.03–2.5], OR = 1.9 [1.9–3.3], and OR = 3 [2–4]), respectively (Table 8). By logistic regression analysis of factors affecting Physical activity, physically inactive cases (N = 186) compared to physically active cases (N = 164) who were residents in urban areas, were not working. In addition, illiterate was more physically inactive compared to patients who live in rural areas, employed and educated patients (OR = 2.3 [1.2–4.6], OR = 2.5 [1.1–5.8], and OR = 3[1.9–4.7]), respectively with significant *P*-value (Table 9).

**Table 2:** Diseases in the studied population.

Comorbid diseases	Cases	Control	*P* value
DM	24 (6.9%)	9 (12.0%)	.131
HTN	50 (14.3%)	9 (12.0%)	.603
DM, HTN	18 (5.1%)	0 (0.0%)	.053
Heart disease	5 (1.4%)	1 (1.3%)	1.000
Chest diseases	14 (14.7%)	0 (0.0%)	.596
Other diseases	2 (0.6%)	0 (0.0%)	
Total	95 (27.1%)	19 (21.3%)	.299

DM = diabetes mellitus, HTN = hypertension.

**Table 3:** Pattern of physical activity in the studied population.

Physical activity	Total (n = 425)	Cases (n = 350)	Control (n = 75)	*P*
Not active	193 (45.4%)	186 (53.1%)	7 (9.3%)	<.001
Active	232 (54.6%)	164 (46.9%)	68 (90.7%)	

**Table 4:** Comparison of the 2 groups according to patterns of activity at work (N = 425).

Pattern of activity at work	Cases (n = 350)	Control (n = 75)	*P*
Vigorous activity			
Yes	92 (26.3%)	15 (20.0%)	.255
No	258 (73.7%)	60 (80.0%)	
(a) Number of days per week	(n = 92)	(n = 15)	
1–3 d	65 (70.7%)	3 (20.0%)	p < .001
4–6 d	23 (25.0%)	12 (80.0%)	
7 d	4 (4.3%)	0 (0.0%)	
Mean ± SD	3.32 ± 1.77	5.13 ± 1.19	<.001
(b) Number of hours per day	(n = 92)	(n = 15)	
Range (maximum–minimal)	10–2	10–4	
Mean ± SD (per min)	296.7 ± 120.5	380.0 ± 95.3	.005
Physical activity per week			
75–150	0 (0%)	0 (0%)	p = .597
150–300	8 (8.7%)	0 (0%)	
>300	84 (91.3%)	15 (100%)	
Mean ± SD	962.6 ± 683.9	1996.0 ± 711.9	<.001
Moderate physical activity			
Yes	135 (38.6%)	55 (73.3%)	<.001
No	215 (61.4%)	20 (26.7%)	
(a) Number of days per week	(n = 135)	(n = 55)	
1–3 d	91 (67.4%)	16 (29.1%)	p < .001
4–6 d	40 (29.6%)	38 (69.1%)	
7 d	4 (3.0%)	1 (1.8%)	
Mean ± SD	3.38 ± 1.74	4.87 ± 1.33	<.001
(b) Number of hours per day	(n = 135)	(n = 55)	
Range (maximum–minimal)	6–1	8–2	
Mean ± SD (per min)	261.8 ± 120.4	355.6 ± 112.5	<.001
Duration of physical activity/week in minutes			
75–150	2 (1.5%)	0 (0.0%)	p = .017
150–300	19 (14.1%)	1 (1.8%)	
>300	114 (84.4%)	54 (98.2%)	
Mean ± SD	900.4 ± 691.4	1825.1 ± 807.7	<.001

MC = Monte Carlo, *U* = Mann–Whitney test.

**Table 5:** Comparison of the studied groups according to pattern of activity during travel from and to places for 10 min.

Pattern of activity during travel to and from places for 10 min	Cases (n = 350)	Control (n = 75)	*P*
Walking to and from places (>10 min)			
Yes No	175 (50.0%)	63 (84.0%)	<.001
	175 (50.0%)	12 (16.0%)	
(a) Number of days/week	(n = 175)	(n = 63)	
Range (maximum–minimal)	7–1	6–1	
Mean ± SD	1.65 ± 1.28	2.56 ± 1.47	<.001
(b) Frequency of hours per day	(n = 175)	(n = 63)	
Range (maximum–minimal)	7–1	6–1	
Mean ± SD (min)	86.40 ± 50.88	129.5 ± 85.67	<.001
Duration of physical activity during travel			
75–150	135 (77.1%)	27 (42.9%)	<.001
150–300	23 (13.1%)	16 (25.4%)	
>300	17 (9.7%)	20 (31.7%)	

**Table 6:** Pattern of recreational (fun) activities in the studied population.

Pattern of recreational activities	Cases (n = 350)	Control (n = 75)	*P*
Vigorous activity			
Yes	53 (15.1%)	7 (9.3%)	.190
No	297 (84.9%)	68 (90.7%)	
(a) Number of days/weeks	(n = 53)	(n = 7)	
Range (maximum–minimal)	3–1	4–1	
Mean ± SD	1.32 ± 0.55	2.14 ± 1.35	.131
(b) Number of hours per day	(n = 53)	(n = 7)	
Range (maximum–minimal)	3–1	4–1	
Mean ± SD (min)	100.8 ± 34.9	171.4 ± 80.71	.015
Recreational physical activity in minutes/week			
75–150	37 (69.8%)	3 (42.9%)	p = .003
150–300	13 (24.5%)	0 (0.0%)	
>300	3 (5.7%)	4 (57.1%)	
Moderate intensity activity			
Yes	12 (3.4%)	8 (10.7%)	p = .014
No	338 (96.6%)	67 (89.3%)	
(a) Number of days/weeks	(n = 12)	(n = 8)	
Range (maximum–minimal)	5–2	5–1	
Mean ± SD	3.25 ± 1.29	2.63 ± 1.51	.305
(b) Number of hours per day	(n = 12)	(n = 8)	
Range (maximum–minimal)	5–2	4–1	
Mean ± SD (min)	215.0 ± 90.3	135.0 ± 62.1	.069
Physical activity			
75–150	0 (0%)	2 (25.0%)	
150–300	3 (25.0%)	3 (37.5%)	
>300	9 (75.0%)	3 (37.5%)	

FE = Fisher exact, *U* = Mann–Whitney test.

**Table 7:** Sedentary behavior pattern in the studied population.

Sedentary behavior	Cases (n = 350)	Control (n = 75)	*P*
Time spent sitting or reclining (except sleeping)			
Yes (>6 h/d)	202 (57.7%)	24 (32.0%)	<.001
No (<6 h/d)	148 (42.3%)	51 (68.0%)	

**Table 8:** Univariate and multivariate logistic regression analysis for factors affecting physical activity in the total sample (n = 193 vs 232) for different parameters.

	Univariate		Multivariate#	
	*P*	OR (LL–UL 95% CI)	*P*	OR (LL–UL 95% CI)
Obese	<.001^*^	11.017 (4.922–24.663)	<.001^*^	11.700 (5.105–26.813)
Sex (female)	.035^*^	1.619 (1.035–2.533)	.373	0.784 (0.459–1.340)
Residence (urban)	.022^*^	1.920 (1.098–3.358)	.024^*^	2.074 (1.099–3.917)
Educational level				
Illiterate	.318	1.912 (0.536–6.820)		
Read and write	.487	0.658 (0.202–2.143)		
Secondary education	.515	0.689 (0.224–2.115)		
Occupational history (not working)	<.001^*^	3.002 (2.007–4.491)	<.001^*^	3.140 (1.972–5.000)

CI = confidence interval, LL = lower limit, OR = odds ratio, ® = Reference group, UL = upper limit.

#All variables.

**Table 9:** Univariate and multivariate logistic regression analysis for factors affecting physical activity in physically inactive cases (N = 186) compared to physically active cases (N = 164) for different parameters.

	Univariate		Multivariate^#^	
	*P*	OR (LL–UL 95% CI)	*P*	OR (LL–UL 95% CI)
Residence (urban)	.010^*^	2.397 (1.236–4.646)	.015^*^	2.360 (1.184–4.703)
Educational level				
Illiterate	.026^*^	2.568 (1.122–5.877)	.323	1.580 (0.637–3.921)
Read and write	.581	1.220 (0.602–2.473)	.855	0.931 (0.433–2.002)
Secondary education	.746	1.098 (0.626–1.926)	.766	1.094 (0.605–1.979)
Occupational history (not working)	<.001^*^	3.048 (1.951–4.763)	<.001^*^	2.812 (1.721–4.594)

CI = confidence interval, LL = lower limit, OR = odds ratio, UL = upper limit.

#All variables.

## 6. Discussion

This current research was designed to characterize the lifestyle of Egyptian individuals in terms of physical activity. Physically active persons and eating a healthy diet are generally established to be essential variables in weight loss for overweight and obese people. Physical exercise is also a component of intervention programs to promote a healthy and active lifestyle.^[16]^

Our study included 350 participants with elevated obese-range BMI and 75 participants with normal-range BMI. The mean age was around 34 years in both groups, the female gender was prominent, the majority were married and were living in a rural village. Additionally, over half of the study group was educated, and more than half of them were working. Our results agreed with a study published in the India journal of community, also agreed with most surveys, references and researches undertaken in Arab nations.^[17,18]^

Regarding the presence of comorbid diseases, around one-quarter of our studied population has chronic diseases (27% of obese patients, 21% in the control group), the most common diseases were hypertension (14%), diabetes, and chest disease, these diseases could be prevented and managed by modifying risk factors such as physical inactivity.^[19]^ Therefore, health promotion and disease-preventive strategies are essential at this time.

Our study showed that more than half of our studied population was physically inactive, and physical inactivity was higher in the obese than the control group (53% and 9%, respectively). Our study results agree with most references and research undertaken in Arab nations which found that obese patients had decreased physical activity due to the burden of weight and comorbidities.^[18]^ In addition, our results found that physical inactivity was consistent with another study in Bangladesh and a study in South America that reported physical inactivity among adults was 50.3% and 44.5%, respectively.^[20]^

In our study, obese patients practiced vigorous physical activity at work more than the control group (26 % vs 20%); however, it was statistically insignificant. Also, the mean number of days per week were less in cases compared to the control group with a statistically significant result (3.32 ± 1.77 in the cases, 5.13 ± 1.19 in control, *P* < .001). Additionally, the mean number of minutes of vigorous activity per day in the study group were less in the case group than the control 1 with a statistically significant value (296.7 ± 120.5, 380.0 ± 95.3, and *P* = .005) agreed with Elabd et al,^[16]^ 2020 who found that obese patients practicing daily physical activity for around 60 minutes were higher than the percentage of nonobese people (68.2% vs 31.8%).

In our study, the pattern of moderate physical activity at work was lower in obese (38%) than the control group (73%), the mean number of days per week were 3.38 ± 1.74 and 4.87 ± 1.33, respectively; and the mean number of hours per day was 261.8 ± 120.4 in control patients and 355.6 ± 112.5 in cases. The results found about the pattern of activity during travel to and from places (10 min) show that obese participants had significantly lower duration of walking to and from places than the control group (50% vs 84%), while the mean number of days per week were less in the case group than the control 1 (1.65 ± 1.28 compared to 2.56 ± 1.47).

Regarding the pattern of recreational activity, it was diminished in the obese patients than the control group; however, it was not statistically significant. In addition, the results agree with Maffeis et al^[21]^ who measured physical activity in a group of obese and normal individuals, finding that nonobese engage in 100 minutes more physical exercise each day than obese individuals.

Sedentary behavior assessment showed that sitting time or reclining (except sleeping) was more significant in obese (57%) than the control participants (*P* < .001). Our results agree with a large-scale study aimed to assess the pattern of physical activity during different times through the day which reported changes in physical activity during different times of the day.^[22]^ Similarly, Scheers et al^[23]^ in a study of 442 adults, found that obese males had more sedentary time than nonobese individuals (+1.09 h/d).

Most studies advised on certain levels of physical activity others focused on leisure time and considered that even low levels of physical activity might have an important public health impact and contribute to improving physical fitness, health-related quality of life, and consequently, overall health.^[24]^

Regarding factors affecting physical activity, in our studied population, obese patients, female gender, persons who were resident in urban areas, and persons who were not working were more physically inactive compared to normal persons (OR = 11 [4.9–24.6], OR = 1.6 [1.03–2.5], OR = 1.9[1.9–3.3], and OR = 3 [2–4], respectively). Our results concur with a study in Singapore which concluded that married females, not working and who had a level of education are physically inactive,^[25]^ this may be due to family responsibilities and child care.

## 7. Strength and limitations

It is a community-based study, and we used a well-standard and validated questionnaire to the assessment of physical activity patterns and sedentary behavior of individuals attending a primary health care unit. There are a few limitations of this study, such as the pattern of physical activity depending on participants’ self-reporting, which could lead to recall bias; also, during the data collection, there was social isolation due to the pandemic.

## 8. Conclusion

Our study found that less than half of the studied population was physically active which is considered low. Physical activity was better among persons with normal BMI compared to obese ones. Obese patients had more prevalence of sedentary life than nonobese. In addition, obese individuals engaged in less recreational activity than normal-weight participants. The residence in urban areas, unemployment status, and low levels of education were independent factors for physical inactivity in patients with obesity. The female gender, illiterate persons, unemployed persons, and obese persons were predicting factors for physical inactivity.

## 9. Recommendation

It is crucial to emphasize the need to educate the whole population, especially the middle-aged population about the value of physical exercise as a cornerstone of a better, healthier life, regardless of their BMI, and come up with suitable strategies to help reduce sedentary behaviors during work times.

## Acknowledgments

We would like to thank all participating patients in this study. We also had the honor of meeting the primary health care unit in El-Mahsma Village, Ismailia, Egypt.

## Author contributions

**Conceptualization:** Almaza A. Salim.

**Data curation:** Almaza A. Salim, Eman Fahmey Nasr.

**Formal analysis:** Noha M. Abu Bakr Elsaid.

**Methodology:** Noha M. Abu Bakr Elsaid

**Software:** Noha M. Abu Bakr Elsaid.

**Supervision:** Mostafa M. Ragheb, Hani Aiash.

**Writing – original draft:** Almaza A. Salim, Eman Fahmey Nasr.

**Writing – review & editing:** Yomna E. Dean, Jose J. Loayza Pintado, Yusef Hazimeh.
